# A Potential Mechanism of Temozolomide Resistance in Glioma–Ferroptosis

**DOI:** 10.3389/fonc.2020.00897

**Published:** 2020-06-23

**Authors:** Zhifang Hu, Yajing Mi, Huiming Qian, Na Guo, Aili Yan, Yuelin Zhang, Xingchun Gao

**Affiliations:** ^1^Shaanxi Key Laboratory of Brain Disorders, School of Basic Medical Science, Xi'an Medical University, Xi'an, China; ^2^Shaanxi Key Laboratory of Ischemic Cardiovascular Disease, Institute of Basic and Translational Medicine, Xi'an Medical University, Xi'an, China

**Keywords:** ferroptosis, glioma, temozolomide, GPX4, drug resistance

## Abstract

Temozolomide (TMZ) is the first-line chemotherapy drug that has been used to treat glioma for over a decade, but the benefits are limited by half of the treated patients who acquired resistance. Studies have shown that glioma TMZ resistance is a complex process with multiple factors, which has not been fully elucidated. Ferroptosis, which is a new type of cell death discovered in recent years, has been reported to play an important role in tumor drug resistance. The present study reviews the relationship between ferroptosis and glioma TMZ resistance, and highlights the role of ferroptosis in glioma TMZ resistance. Finally, the investigators discussed the future orientation for ferroptosis in glioma TMZ resistance, in order to promote the clinical use of ferroptosis induction in glioma treatment.

## Introduction

Gliomas originate in the neuroectodermal layer, and are the most common CNS tumors in clinic. This accounts for more than 30% of all primary intracranial tumors, and 74.6% of these are malignant gliomas (1). The present treatment of gliomas is based on surgical treatment, combined with radiotherapy and chemotherapy. Due to the specificity of the anatomical locations of gliomas and the characteristics of invasive growth, and the toilless relapse after an operation, the median survival of high-grade glioma patients is only 12–15 months, and the 5-year survival rate is <5% (2).

Clinical practice has proven that chemotherapy can effectively improve the survival time and rate of glioma patients in the comprehensive treatment of glioma. Among the first-line chemotherapeutic drugs of gliomas, temozolomide (TMZ) has been considered to be the most effective treatment drug for glioma due to its advantages, such as oral administration, easy penetration through the blood-brain barrier, acidic environment stability, and no toxicity of superposition with other drugs. Studies have shown that postoperative adjuvant TMZ chemotherapy can increase the survival period from 12.1 months to 14.6 months in high-grade glioma patients (3). This 2.5-month survival period extension is a huge improvement in treatment. However, in clinical applications, the efficacy of TMZ was found to be only 46%, and this was even lower in recurrent glioma patients (4). The glioma resistance to TMZ is the most important cause of chemotherapy failure. Studies have shown that glioma TMZ resistance is the result of multiple factors, and the molecular mechanisms are very complex.

In 2012, Dixon et al. (5) investigated the mechanism of selective killing of RAS-mutated tumor cells through the anti-tumor drug erastin. Then, they discovered and reported a new cell death mode-ferroptosis, which has great differences in morphology, biochemistry and genetic aspects with the presently known apoptosis, necrosis, and autophagic cell death. A number of studies have revealed that ferroptosis plays an important role in cancer development and drug resistance (6, 7). A clear understanding of ferroptosis in glioma TMZ resistance would benefit the clinical practice of applying ferroptosis to glioma therapy. The present review summarizes the mechanisms of ferroptosis, and the signaling pathways involved in ferroptosis and glioma TMZ resistance.

## Ferroptosis

**Figure 1:**
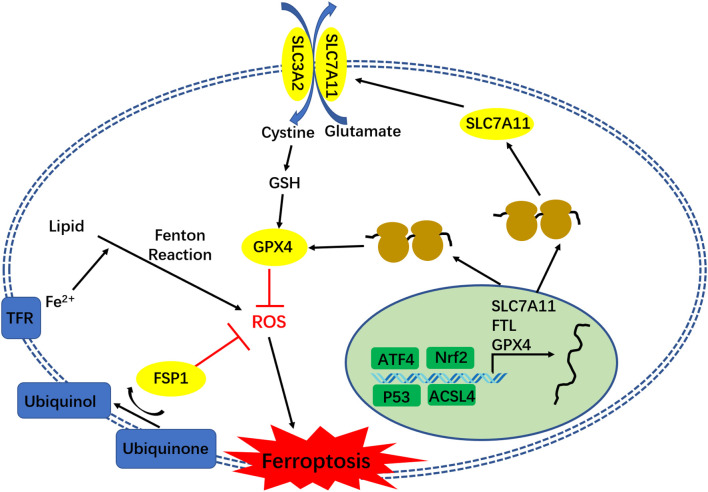
The regulatory network of ferroptosis.

Ferroptosis is distinct from other forms of regulated cell death, such as apoptosis, autophagy, necroptosis, and pyroptosis (8). The typical signs of ferroptosis are the increase in cellular lipid reactive oxygen, shrunken mitochondria, and the increase in mitochondrial membrane density (5). Studies have found that iron-dependent Fenton reaction and glutathione (GSH) loss, which lead to reactive oxygen species (ROS) accumulation in cells, are the direct causes of ferroptosis. When the iron homeostasis in tissue cells is disrupted, the excess iron would be converted to H_2_O_2_ and lipid peroxides would be converted into ROS via the Fenton reaction, which induces ferroptosis. However, this can be specifically reversed by iron chelators (9). Cysteine (Cys) is the rate-limiting substrate for GSH synthesis. The Cys uptake in cells is regulated by the cystine/glutamate reverse transporter functional subunit Solute Carrier Family 7 Member 11 (SLC7A11). Ferroptosis inducer erastin induces ferroptosis by inhibiting SLC7A11 from blocking Cys absorption, and reducing GSH synthesis to promote ROS accumulation (5). The decrease in GSH content in cells can also inhibit the activity of Glutathione peroxidase 4 (GPX4), resulting in the decrease in cell antioxidant capacity and lipid reactive oxygen increase, and eventually causing ferroptosis (10, 11). Recently, researchers have found that ferroptosis suppressor protein 1 (FSP1) (previously known as apoptosis-inducing factor mitochondrial 2 [AIFM2]) is a potent ferroptosis resistance factor, which has a protective effect on GPX4 deletion-induced ferroptosis (12, 13). Studies conducted over the past decade have defined core regulators that regulated cell ferroptosis, including GPX4, nuclear factor erythroid 2-related factor 2 (nrf2), SLC7A11, Activated transcription factor 4 (ATF4), p53 (especially acetylation-defective mutant p53) (Figure 1), and FSP1. All these regulators also play an important role in glioma TMZ resistance. The present study will review the relationship between ferroptosis and glioma TMZ resistance, and highlight the role of ferroptosis in glioma TMZ resistance.

## Temozolomide Resistance in Glioma

The chemical name of TMZ is 3-methyl-4-oxoimidazo[5,1-d](1–3, 5)tetrazine-8-carboxamide. This is an oral alkylating agent, and its antitumor activity was first discovered in 1987 (14). This became the effective first-line chemotherapeutic agent for the treatment of glioma patients since the FDA approved its efficacy in 2005 (15, 16). TMZ elicits cytotoxicity through the methylation of DNA guanine residues at the O6 and N7 positions, and adenine at the N3 position (17). The alkylation of the O6 site on guanine leads to its subsequent nucleotide mispairing with thymine instead of cytosine during the subsequent DNA replication (18). Then, the mismatched lethal base pairs would cause single- and double-strand DNA breaks, induce cell cycle arrest at the G2/M, and eventually lead to cell death (19, 20). As it is known, the methylated DNA can be repaired by methyltransferase, and O6-methylguanine methyltransferase (MGMT) can reverse methylation of the O6 position of guanine. Hence, many studies have proven that glioma patients with high MGMT expression are naturally resistant to TMZ (21, 22). In addition to the natural TMZ resistance, glioma patients can obtain TMZ resistance through a variety of mechanisms when treated with TMZ. Recent advances in the understanding glioma TMZ resistance have introduced presented many novel independent mechanisms, such as epigenetic regulation for transcription, including miRNA (23), histone modification (24, 25), and DNA methylation (26). Autophagy is a mechanism to maintain cell homeostasis, which is activated in tumor cells through radiation and chemotherapeutic agents (27, 28). Furthermore, a number of studies have found that TMZ treatment could induce autophagy to help glioma cells resist the TMZ treatment (16, 29). Apoptosis is the final common pathway triggered by TMZ cytotoxicity (30), and the caspase and Bcl-2 family members all regulate glioma TMZ resistance (30–32). In addition, autophagy also takes part in the TMZ-induced cytotoxicity for glioma cells, and targeting autophagy can be sensitive to TMZ treatment (29). Evidence suggests that glioma stem cells (GSC) is a subpopulation of glioblastoma cells, which are capable of self-renewal, tumorigenesis, radio-resistance, and chemo-resistance (including TMZ) (33–35). Recent studies have shown that ferroptosis takes part in glioma TMZ resistance, which may be a new potential mechanism for TMZ resistance in glioma. This will be discussed in detail below.

## Ferroptosis and Glioma Temozolomide Resistance

### The Role of Iron Metabolism in Ferroptosis and TMZ Resistance

Ferroptosis is an iron dependent form of programmed cell death, and the excess iron contributes to ferroptosis by producing ROS through Fenton reaction (36). Iron is an inorganic element for cell basic function, and is especially highly required for cancer cells (37). The iron metabolism is a complex network of iron absorption, storage, and export. Transferrin receptor (TfR1) binds ferric iron (Fe3+) in circulation, and binds to the membrane protein transferrin receptor (TfR). Then, the Tf-Fe-TfR conjugate enters the cell through clathrin-mediated endocytosis (38). Cytoplasmic iron is stored in ferritin, which is an iron storage protein complex that consists of ferritin light chain (FTL) and ferritin heavy chain 1 (FTH1). Finally, the excess iron is exported by the membrane protein ferroportin (an iron efflux pump, also termed as SLC11A3) (39).

Many studies have shown that iron is an essential element for drug-resistant cells. Hence, ferroptosis, which is an iron-related cell death, also plays an important role in cancer drug-resistance. Schonberg et al. (40) reported that the expression of transferrin is upregulated in GSC, and that the iron uptake promoted the GSC proliferation, while disrupting the iron metabolism in GSCs could reduce the GSC cell growth (41). Multiple studies have revealed that the expression of TfR1 and TfR2 are upregulated in proliferating and malignant cells, including GBM (42, 43). The study conducted by Calzolari et al. has proven that TfR2 expression is inversely correlated with tumor histologic grade, and is associated with the sensitivity to TMZ of glioma cells (43, 44). Feng et al. (45) reported that TfR is a specific ferroptosis maker. These results show that iron metabolism is involved in glioma TMZ resistance and ferroptosis. Furthermore, the study conducted by Chen et al. (46) has proven that erastin (ferroptosis inducer) sensitizes glioma cells to TMZ. Iron-chelating agents (ferroptosis inhibitor), which are used to remove excess iron by binding free iron, can enhance the TMZ cytotoxicity in glioma cells. The study conducted by Alexiou et al. (47) has proven that deferiprone (DFP), which is an orally administered iron chelator, can significantly reduce glioma cell viability combination with TMZ. It has been reported that curcumin, which is a biologically active iron chelator (48, 49), can sensitizes glioma to TMZ by simultaneously generating ROS and disrupting the AKT/mTOR signaling (50). Recently, Chen et al. (51) reported that ALZ003, which is a curcumin analog, induces ferroptosis by blocking the AR-mediated GPX4 expression, inhibiting TMZ–resistant glioblastoma.

### ROS in Ferroptosis and TMZ Resistance

ROS is produced by metabolic pathways, and play important roles in tissue homeostasis and cell signaling (52, 53). The iron-dependent accumulation of ROS is the characteristic of ferroptosis (54). Furthermore, ROS-induced lipid peroxidation is not only a critical role in ferroptosis, but also affects the apoptosis, autophagy, and TMZ resistance in glioma cells (53, 55, 56). Biochemically, the intracellular GSH depletion, which inhibits anti-peroxidant defense and iron, involved in the Fenton reaction would result to the accumulation of lipid peroxidation, promoting ferroptosis. The present study will summarize the lipid peroxidation regulating mechanism between ferroptosis and glioma TMZ resistance.

#### SLC7A11 Regulates Ferroptosis in Glioma TMZ Resistance

SLC7A11, which is also known as xCT, encodes 12 transmembrane domains, and is a functional subunit that constitutes the cystine/glutamate counter transporter (System Xc-) that regulates the intracellular glutamate and extracellular cystine exchange. Cysteine is the rate-limiting substrate for the synthesis of biological antioxidant glutathione (GSH). Sufficient cystine can ensure the synthesis of intracellular GSH, and the level of GSH in cells plays an important role in glioma drug resistance (57). Chen et al. reported that highly expressed SLC7A11 in gliomas can regulate the resistance of TMZ. However, suppressing the SLC7A11 expression can enhance the sensitivity of gliomas to TMZ using siRNA (46). Polewski et al. (58) also confirmed that the inhibition of SLC7A11 expression in gliomas can reduce the level of GSH in cells, and increase the sensitivity to TMZ.

The latest research shows that SLC7A11 plays an important role in the regulation of ferroptosis. SLC7A11 can participate in ferroptosis by regulating the synthesis of GSH, affecting the level of ROS in cells, and the accumulation of lipid peroxides. Studies have shown that salicylic acid sulfapyridine (SAS) inhibits SLC7A11 activity, which can cause ferroptosis and increase the cystine intake using β-mercaptoethanol, in order to reverse the ferroptosis inducer erastin-induced ferroptosis in HT1080 cells (5). Jiang et al. (59) reported that the overexpression of SLC7A11 in tumor cells inhibits ROS-induced ferroptosis, and weakens the inhibitory effect of p53 3KR on tumor growth. In the SLC7A11 knockout mouse model, SLCA11 was confirmed to inhibit the cell ferroptosis induced by iron overload (60). The toxicity of TMZ to glioma cells is correlated to the expression of SLC7A11, and ferroptosis would enhance this effect of TMZ (61). These findings suggest that SLC7A11 may play an important role in the TMZ resistance of glioma by regulating ferroptosis.

#### The Effect of GPX4 in Ferroptosis and TMZ Resistance

GPX4 is a powerful antioxidant enzyme in the body. GSH is used as a substrate to reduce lipid oxidation products, and suppress the ferroptosis. The ferroptosis inducer RSL3 mainly induces ferroptosis by inhibiting the activity of GPX4. The expression of GPX4 in gliomas increases with the increasing grades of gliomas, and the expression of GPX suppressed by siRNA can inhibit the proliferation and migration of glioma cells (62). Further studies have found that GPX4 plays an important role in tumor resistance. Inhibiting the activity of GPX4 can enhance the ferroptosis of drug-resistant cells, and thereby enhance the sensitivity to chemotherapeutics (63). The treatment of glioma cells with the GPX4 inhibitor RSL3 can enhance the ferroptosis of cells (64). All these studies suggest that GPX4 may play an important role in the resistance of tumor cells by regulating ferroptosis, including glioma TMZ resistance, but the regulatory mechanisms need further study.

#### The Effect of ACSL4 in Ferroptosis and TMZ Resistance

Yang et al. (65) reported that polyunsaturated fatty acids (PUFAs) are the most susceptible lipids to ROS damage in the course of ferroptosis, when compared to other the classes of lipids by lipidomics analysis. Acyl-CoA synthetase long-chain family member 4 (ACSL4) is a member of the long chain family of acyl-CoA synthetase proteins, and an enzyme responsible for esterifying polyunsaturated fatty acids (PUFA) into acyl CoA, which is a necessary step to form PUFA-containing phospholipids (66, 67). Yuan et al. (67) reported that ACSL4 contributes to ferroptosis by producing of 5-hydroxyeicosatetraenoic acid (5-HETE). The study conducted by Doll et al. has also proven that the inhibition of ACSL4 expression could decrease the oxidization of a number of sensitive fatty acids (arachidonic acid [AA] and AdA-containing PE species) in the membrane, as critical determinants of sensitivity to ferroptosis. Furthermore, the pharmacological inhibition of ACSL4 (such as thiazolidinediones [TZD]) could prevent ferroptosis (66). Recently, Cheng et al. reported that the expression of ACSL4 was downregulated in human glioma tissues and cells. The overexpression of ACSL4 decreased the expression of GPX4, increased the levels of 5-HETE, and induced a reduction in cell viability. The siRNA-mediated silencing of ACSL4 promoted the proliferation in glioma cells via the decrease in ferroptosis through the enhancement of GPX4 expression. All these results suggest that ACSL4 inhibition directly affects the expression of GPx4, which finally promotes glioma cell proliferation by inhibiting ferroptosis (68). As mentioned above, GPX4 plays an important role in the resistance of tumor cells by regulating ferroptosis. Therefore, ACSL4 may also regulate TMZ resistance in gliomas by affecting the GPX4 expression. However, the study of its resistance to TMZ in gliomas has not been reported at present.

### Other Pathways Regulate Ferroptosis and Glioma TMZ Resistance

#### Nrf2 Regulates Ferroptosis in Glioma TMZ Resistance

Nrf2 is an important transcription factor that regulates the antioxidant stress response in cells, and is mainly combined with the antioxidant response element (ARE) to regulate downstream antioxidant protein expression, and respond to oxidative stress. In Nrf2 knockout mice, it was found that Nrf2 appears with two different effects on the occurrence and development of tumors: under physiological conditions, it can protect normal cells and suppress tumorigenesis; however, it has a “dark” side in the pathophysiological process of tumorigenesis and development. Nrf2 and its downstream genes are highly expressed in many tumor cells (including gliomas) or tissues, and it has a role in promoting tumor growth, proliferation, and drug resistance (69–72). Numerous studies have shown that Nrf2 also plays an important role in TMZ resistance in gliomas (73–75). The treatment of glioma cells with TMZ would induce high expression of Nrf2, and the inhibition of Nrf2 expression can increase the sensitivity of glioma cells to TMZ (73–75).

Many researches have shown that Nrf2 also plays an important role in the regulation of ferroptosis. Kerins et al. reported that several genes (FTL/FTH1, FPN1, and GPX4) involved in iron storage and iron ion export are regulated by Nrf2. Hence, Nrf2 can affect cell iron stability and regulate iron-dependent cell death-ferroptosis (76). Another study further pointed out that Nrf2 is a negative regulator of ferroptosis, and that the ferroptosis inducer erastin can inhibit the degradation of Nrf2 by regulating the p62-Keap1-NRF2 signal pathway, thereby inhibiting the occurrence of ferroptosis in hepatoma cells (77). The inhibition of the Nrf2-ARE pathway could reverse the resistance to drugs and ferroptosis in head-neck cancer cells (78). Patients with highly expressed Nrf2 in gliomas have poor prognosis and a short survival time (64). The overexpression of Nrf2 can promote the proliferation of glioma cells. Further studies have found that Nrf2 can regulate the expression of SLC7A11 to resist ferroptosis (64). Wang et al. also demonstrated that iron can regulates SLC7A11 transcription through ROS-Nrf2-ARE, thereby affecting the occurrence of ferroptosis in liver cells (60). These above findings indicate that Nrf2 can resist ferroptosis by regulating the expression of SLC7A11, which could affect the TMZ resistance of glioma by regulating ferroptosis.

#### ATF4 Regulates Ferroptosis in Glioma TMZ Resistance

Activated transcription factor 4 (ATF4) is a basic leucine zipper transcription factor that belongs to the cAMP response element binding protein (CREB) family, which is also known as cAMP-response element binding protein2 (CREB2). This regulates the downstream gene transcription by binding an amino acid response element (AARE) in the promoter of the target gene. ATF4 is involved in regulating a variety of physiological and pathological processes, such as the regulation of hematopoiesis, osteoblast differentiation, endoplasmic reticulum stress, and tumor growth. Previous studies have reported that the promoter region of SLC7A11 contains two AARE elements, which is a direct downstream gene of ATF4. ATF4 in bladder cancer cells can regulate the expression of SLC7A11 (79). Studies have shown that glioma patients with a high expression of ATF4 have a shorter survival time, and ATF4 can inhibit the ferroptosis of glioma cells by regulating the expression of the downstream gene SLC7A11, in order to promote tumor proliferation and angiogenesis (80). More recently, Chen et al. (81) reported that Dihydroartemisinin (DHA) could induce ferroptosis in glioma cells through the PERK/ATF4/HSPA5 pathway. Further research has revealed that the TMZ treatment of glioma cells led to the increase in ATF4 expression, the overexpression of ATF4 can enhance the glioma cells resistance to TMZ, and the inhibition of ATF4 expression can increase the sensitivity of glioma cells to TMZ. Furthermore, the ATF4 promotion to glioma TMZ resistance was achieved by enhancing the expression of SLC7A11 (82). In brief, these findings indicate that ATF4 can affect glioma ferroptosis and TMZ resistance by regulating the expression of SLC7A11.

#### Effect of P53 in Ferroptosis and TMZ Resistance

P53 is one of the most extensively studied tumor suppressor gene, and was named for encoding a 53 KD protein. Normal P53 in cells can regulate cell proliferation, DNA damage repair, senescence, and apoptosis, etc. When P53 mutated, it loses the function of regulation of cell growth, and becomes an oncogene. Studies have shown that P53 also plays an important role in TMZ resistance in gliomas. Lee et al. reported that the effect of TMZ and chloroquine in the treatment of gliomas depend on the p53 condition, and that the combination therapy can inhibit the proliferation of P53-positive glioma cells and the promotion of apoptosis. However, this has no effect on glioma cells with mutant P53 (83). Further studies have found that mutations and deletions in the P53 gene frequently occur in gliomas. Another research revealed that 28% of glioma patients had P53 mutations (84), while P53 mutations are considered to have a negative influence on glioma radiotherapy and TMZ therapy (85, 86). P53 mutation is closely correlated to the poor prognosis of patients with glioblastoma, and may reduce the sensitivity of glioblastoma to TMZ by increasing the expression of drug resistance gene MGMT (87).

Recent studies have shown that P53 also plays an important role in the regulation of ferroptosis. P53 directly targets SLC7A11, and reduces the cell uptake of cystine by inhibiting the transcription of SLC7A11, thereby limiting the production of GSH in cells, significantly increasing the sensitivity of ferroptosis, and further discovering an acetylation defect p53 3KR mutant. Hence, this loses its ability to induce cell cycle arrest, apoptosis, and aging, but retains the ability to inhibit ferroptosis through the suppression of SLC7A11 (59). P53 can slow down the consumption of GSH in cells and reduce ROS production by regulating the p53-p21 axis, and inhibit ferroptosis due to the metabolic pressure (88). The above-mentioned researches indicate that P53 plays an important role in both TMZ resistance and ferroptosis in gliomas, but the specific regulatory mechanisms still need further studies and elucidation.

### The Relationship Among Ferroptosis, Autophagy, Apoptosis, and Glioma TMZ Resistance

As mentioned above, autophagy and apoptosis also play important role in glioma TMZ resistance. An original study reported that ferroptosis is distinct from other regulated cell deaths (RCDs), including autophagy, apoptosis, and necrosis (5). However, recently, numerous studies have revealed that there is a complex relationship between ferroptosis and other types of cell death, including autophagy and apoptosis (89). Hou et al. (90) reported that the activation of the autophagy pathway promotes ferroptosis through the degradation of ferritin. Torii et al. (91) suggested that autophagy contributes to erastin-induced ferroptosis through the generation of lysosomal ROS. Yang et al. (92) reported that autophagy promotes ferroptosis by regulating the novel ARNTL-EGLN1-HIF1A pathway. All these suggest that the activation of the autophagic machinery can trigger ferroptosis, and ferroptosis inducers enhance the cell death by activating the autophagy (89, 93). Considering that autophagy also takes part in glioma TMZ resistance (20), the inhibition of autophagy can enhance the TMZ cytotoxicity to glioma cells (94). These suggest that ferroptosis may play an important role in glioma TMZ resistance. Consistently, a recent study also revealed that the inhibition of autophagy could increase the susceptibility of GSC to TMZ by igniting ferroptosis (95).

Many researches have shown that glioma cells undergo apoptosis after treatment with TMZ (30). Recently, researchers found an interrelationship between ferroptosis and apoptosis, and that ferroptosis may occur while sharing common signals or regulators with apoptosis. Zheng et al. (96) reported that nanomaterial MON-p53 can eradicate cancer cells by switching apoptosis to ferroptosis. Hong et al. suggested that ferroptotic agent-induced ER stress response plays an important role in the cross-talk between ferroptosis and apoptosis (97, 98). Furthermore, lipid peroxidation not only leads to ferroptosis, but also stimulates the activation of both the intrinsic and extrinsic apoptotic signaling pathways (53). All these suggest that ferroptosis may regulate glioma TMZ resistance by affecting the autophagy or apoptosis. However, the mechanism needs further research.

## Conclusion and Perspective

The mechanism of gliomas drug resistance to TMZ is very complex, and is not fully understood. The present understanding cannot explain all drug resistance phenomena, and the present status of TMZ drug resistance has not improved in clinical gliomas. Ferroptosis is a newly discovered death mode, which plays an important role in the TMZ resistance of gliomas, and ferroptosis resistance may be a new mechanism of TMZ resistance in gliomas. Targeted ferroptosis can be used as one of the potential therapies to reverse TMZ resistance. This would be the multi-cytotoxic strategy, in which ferroptosis inducers or xCT inhibitors combination with TMZ are used to treat glioma patients. As a chemo-sensitizer by ferroptosis-induction, erastin could sensitize glioblastoma cells to temozolomide (46). A curcumin analog (ALZ003) could induce the TMZ-resistant glioma cell growth ferroptosis by disrupting the GPX4-Mediated redox homeostasis (51). This suggests that GPX4 and alter ROS could be novel targets for developing anti-cancer drugs. Although the advantages of ferroptosis are promising in cancer treatment, there are still details that needs to be formally addressed in the pre-clinical setting and clinical achievability, and this is partly due to the complexity observed in different contexts, such as P53 or Ras-mutant cancer cells (8). Hence, further studies on ferroptosis mechanism in the TMZ resistance of gliomas would provide new ideas and new targets for the clinical reversal of drug resistance in glioma TMZ chemotherapy.

## Author Contributions

ZH and HQ produced the manuscript. YZ and XG conceived and designed framework of this article. ZH and NG collected and analyzed the literature. All authors contributed to the article and approved the submitted version.

## Conflict of Interest

The authors declare that the research was conducted in the absence of any commercial or financial relationships that could be construed as a potential conflict of interest.

## References

[1] Ostrom QT Gittleman H Xu J Kromer C Wolinsky Y Kruchko C. CBTRUS statistical report: primary brain and other central nervous system tumors diagnosed in the United States in 2009-2013. Neuro Oncol. (2016) 18:v1–v75. 10.1093/neuonc/now207 28475809 PMC8483569

[2] Khasraw M Lassman AB. Advances in the treatment of malignant gliomas. Curr Oncol Rep. (2010) 1:26–33. 10.1007/s11912-009-0077-4 20425605

[3] Thomas AA Brennan CW DeAngelis LM Omuro AM. Emerging therapies for glioblastoma. JAMA Neurol. (2014) 11:1437–44. 10.1001/jamaneurol.2014.1701 25244650

[4] Garcia M Clopes A Bruna J Martinez M Fort E Gil M. Critical appraisal of temozolomide formulations in the treatment of primary brain tumors: patient considerations. Cancer Manage Res. (2009) 2009:137–150. 10.2147/CMAR.S5598 21188132 PMC3004664

[5] Dixon SJ Lemberg KM Lamprecht MR Skouta R Zaitsev EM Gleason CE Ferroptosis: an iron-dependent form of nonapoptotic cell death. Cell. (2012) 5:1060–72. 10.1016/j.cell.2012.03.042 PMC3367386 22632970

[6] Friedmann Angeli JP Krysko DV Conrad M. Ferroptosis at the crossroads of cancer-acquired drug resistance and immune evasion. Nat Rev Cancer. (2019) 7:405–14. 10.1038/s41568-019-0149-1 31101865

[7] Gao X Guo N Xu H Pan T Lei H Yan A. Ibuprofen induces ferroptosis of glioblastoma cells via downregulation of nuclear factor erythroid 2-related factor 2 signaling pathway. Anticancer Drugs. (2020) 31:27–34. 10.1097/CAD.0000000000000825 31490283

[8] Mou Y Wang J Wu J He D Zhang C Duan C. Ferroptosis, a new form of cell death: opportunities and challenges in cancer. J Hematol Oncol. (2019) 1:34. 10.1186/s13045-019-0720-y 30925886 PMC6441206

[9] Xie Y Hou W Song X Yu Y Huang J Sun X. Ferroptosis: process and function. Cell Death Differ. (2016) 3:369–79. 10.1038/cdd.2015.158 26794443 PMC5072448

[10] Yang WS Sri Ramaratnam R Welsch ME Shimada K Skouta R Viswanathan VS. Regulation of ferroptotic cancer cell death by GPX4. Cell. (2014) 1–2:317–31. 10.1016/j.cell.2013.12.010 24439385 PMC4076414

[11] Dixon SJ Stockwell BR The hallmarks of ferroptosis. Annu Rev Canc Biol. (2019) 3:35–54. 10.1146/annurev-cancerbio-030518-055844 PMC12851585 41613499

[12] Bersuker K Hendricks JM Li Z Magtanong L Ford B Tang PH. The CoQ oxidoreductase FSP1 acts parallel to GPX4 to inhibit ferroptosis. Nature. (2019) 7784:688–92. 10.1038/s41586-019-1705-2 31634900 PMC6883167

[13] Doll S Freitas FP Shah R Aldrovandi M da Silva MC Ingold I. FSP1 is a glutathione-independent ferroptosis suppressor. Nature. (2019) 7784:693–8. 10.1038/s41586-019-1707-0 31634899

[14] Stevens MF Hickman JA Langdon SP Chubb D Vickers L Stone R. Antitumor activity and pharmacokinetics in mice of 8-carbamoyl-3-methyl-imidazo[5,1-d]-1,2,3,5-tetrazin-4(3H)-one (CCRG 81045; M & B 39831), a novel drug with potential as an alternative to dacarbazine. Cancer Res. (1987) 22:5846–52. 3664486

[15] Stupp R Mason WP van den Bent MJ Weller M Fisher B Taphoorn MJ Radiotherapy plus concomitant and adjuvant temozolomide for glioblastoma. N Engl J Med. (2005) 10:987–96. 10.1056/NEJMoa043330 15758009

[16] Jiapaer S Furuta T Tanaka S Kitabayashi T Nakada M. Potential strategies overcoming the temozolomide resistance for glioblastoma. Neurol Med Chir (Tokyo). (2018) 10:405–21. 10.2176/nmc.ra.2018-0141 30249919 PMC6186761

[17] Denny BJ Wheelhouse RT Stevens MF Tsang LL Slack JA. NMR and molecular modeling investigation of the mechanism of activation of the antitumor drug temozolomide and its interaction with DNA. Biochemistry. (1994) 31:9045–51. 10.1021/bi00197a003 8049205

[18] Kanzawa T Bedwell J Kondo Y Kondo S Germano IM. Inhibition of DNA repair for sensitizing resistant glioma cells to temozolomide. J Neurosurg. (2003) 6:1047–52. 10.3171/jns.2003.99.6.1047 14705733

[19] Lee SY Temozolomide resistance in glioblastoma multiforme. Genes Dis. (2016) 3:198–210. 10.1016/j.gendis.2016.04.007 30258889 PMC6150109

[20] Kanzawa T Germano IM Komata T Ito H Kondo Y Kondo S. Role of autophagy in temozolomide-induced cytotoxicity for malignant glioma cells. Cell Death Differ. (2004) 4:448–57. 10.1038/sj.cdd.4401359 14713959

[21] van Nifterik KA van den Berg J van der Meide WF Ameziane N Wedekind LE Steenbergen RDM. Absence of the MGMT protein as well as methylation of the MGMT promoter predict the sensitivity for temozolomide. Br J Cancer. (2010) 1:29–35. 10.1038/sj.bjc.6605712 20517307 PMC2905289

[22] Donson AM Addo-Yobo SO Handler MH Gore L Foreman NK. MGMT promoter methylation correlates with survival benefit and sensitivity to temozolomide in pediatric glioblastoma. Pediatr Blood Cancer. (2007) 4:403–7. 10.1002/pbc.20803 16609952

[23] Li Y Liu Y Ren J Deng S Yi G Guo M. miR-1268a regulates ABCC1 expression to mediate temozolomide resistance in glioblastoma. J Neuro Oncol. (2018) 3:499–508. 10.1007/s11060-018-2835-3 29876787

[24] Banelli B Carra E Barbieri F Wurth R Parodi F Pattarozzi A. The histone demethylase KDM5A is a key factor for the resistance to temozolomide in glioblastoma. Cell Cycle. (2015) 21:3418–29. 10.1080/15384101.2015.1090063 26566863 PMC4825557

[25] Abe H Natsumeda M Kanemaru Y Watanabe J Tsukamoto Y Okada M. MGMT expression contributes to temozolomide resistance in H3K27M-mutant diffuse midline gliomas and MGMT silencing to temozolomide sensitivity in IDH-mutant gliomas. Neurol Med Chir. (2018) 7:290–5. 10.2176/nmc.ra.2018-0044 29848907 PMC6048353

[26] Chai RC Chang YZ Wang QW Zhang KN Li JJ Huang H. A novel DNA methylation-based signature can predict the responses of MGMT promoter unmethylated glioblastomas to temozolomide. Front Genet. (2019) 10:910. 10.3389/fgene.2019.00910 31611911 PMC6776832

[27] Sui X Chen R Wang Z Huang Z Kong N Zhang M. Autophagy and chemotherapy resistance: a promising therapeutic target for cancer treatment. Cell Death Dis. (2013) 4:e838. 10.1038/cddis.2013.350 24113172 PMC3824660

[28] Gewirtz DA. The challenge of developing autophagy inhibition as a therapeutic strategy. Cancer Res. (2016) 19:5610–4. 10.1158/0008-5472.CAN-16-0722 27634767 PMC5050139

[29] Yan Y Xu Z Dai S Qian L Sun L Gong Z. Targeting autophagy to sensitive glioma to temozolomide treatment. J Exp Clin Cancer Res. (2016) 35:23. 10.1186/s13046-016-0303-5 26830677 PMC4736617

[30] Roos WP Batista LF Naumann SC Wick W Weller M Menck CF. Apoptosis in malignant glioma cells triggered by the temozolomide-induced DNA lesion O6-methylguanine. Oncogene. (2007) 2:186–97. 10.1038/sj.onc.1209785 16819506

[31] Li G Zhang H Liu Y Kong L Guo Q Jin F. Effect of temozolomide on livin and caspase-3 in U251 glioma stem cells. Exp Ther Med. (2015) 3:744–50. 10.3892/etm.2014.2144 25667622 PMC4316973

[32] Gratas C Sery Q Rabe M Oliver L Vallette FM Bak and Mcl-1 are essential for Temozolomide induced cell death in human glioma. Oncotarget. (2014) 9:2428–35. 10.18632/oncotarget.1642 PMC4058016 24811082

[33] Bao S Wu Q McLendon RE Hao Y Shi Q Hjelmeland AB Glioma stem cells promote radioresistance by preferential activation of the DNA damage response. Nature. (2006) 7120:756–60. 10.1038/nature05236 17051156

[34] Lathia JD Mack SC Mulkearns-Hubert EE Valentim CL Rich JN Cancer stem cells in glioblastoma. Genes Dev. (2015) 12:1203–17. 10.1101/gad.261982.115 PMC4495393 26109046

[35] Fukushima T Takeshima H Kataoka H. Anti-glioma therapy with temozolomide and status of the DNA-repair gene MGMT. Anticancer Res. (2009) 11:4845–54. 20032445

[36] Wang S Luo J Zhang Z Dong D Shen Y Fang Y. Iron and magnetic: new research direction of the ferroptosis-based cancer therapy. Am J Cancer Res. (2018) 10:1933–46. 30416846 PMC6220147

[37] Wang Y Yu L Ding J Chen Y Iron metabolism in cancer. Int J Mol Sci. (2019) 20:95 10.3390/ijms20010095 PMC6337236 30591630

[38] Mayle KM Le AM Kamei DT. The intracellular trafficking pathway of transferrin. Biochim Biophys Acta. (2012) 3:264–81. 10.1016/j.bbagen.2011.09.009 21968002 PMC3288267

[39] Kazan HH Urfali-Mamatoglu C Gunduz U. Iron metabolism and drug resistance in cancer. Biometals. (2017) 5:629–41. 10.1007/s10534-017-0037-7 28766192

[40] Schonberg DL Miller TE Wu Q Flavahan WA Das NK Hale JS. Preferential iron trafficking characterizes glioblastoma stem-like cells. Cancer Cell. (2015) 4:441–55. 10.1016/j.ccell.2015.09.002 26461092 PMC4646058

[41] Legendre C Garcion E. Iron metabolism: a double-edged sword in the resistance of glioblastoma to therapies. Trends Endocrinol Metab. (2015) 6:322–31. 10.1016/j.tem.2015.03.008 25936466

[42] Recht L Torres CO Smith TW Raso V Griffin TW. Transferrin receptor in normal and neoplastic brain tissue: implications for brain-tumor immunotherapy. J Neurosurg. (1990) 6:941–5. 10.3171/jns.1990.72.6.0941 2159987

[43] Voth B Nagasawa DT Pelargos PE Chung LK Ung N Gopen Q. Transferrin receptors and glioblastoma multiforme: current findings and potential for treatment. J Clin Neurosci. (2015) 7:1071–6. 10.1016/j.jocn.2015.02.002 25891893

[44] Calzolari A Larocca LM Deaglio S Finisguerra V Boe A Raggi C. Transferrin receptor 2 is frequently and highly expressed in glioblastomas. Transl Oncol. (2010) 2:123–34. 10.1593/tlo.09274 20360937 PMC2847320

[45] Feng H Schorpp K Jin J Yozwiak CE Hoffstrom BG Decker AM. Transferrin receptor is a specific ferroptosis marker. Cell Rep. (2020) 10:3411–23.e3417. 10.1016/j.celrep.2020.02.049 32160546 PMC7172030

[46] Chen L Li X Liu L Yu B Xue Y Liu Y. Erastin sensitizes glioblastoma cells to temozolomide by restraining xCT and cystathionine-gamma-lyase function. Oncol Rep. (2015) 3:1465–74. 10.3892/or.2015.3712 25585997

[47] Alexiou GA Gerogianni P Vartholomatos E Kyritsis AP. Deferiprone enhances temozolomide cytotoxicity in glioma cells. Cancer Invest. (2016) 10:489–95. 10.1080/07357907.2016.1233424 27768402

[48] Jiao Y Wilkinson J 4th Di X Wang W Hatcher H Kock ND. Curcumin, a cancer chemopreventive and chemotherapeutic agent, is a biologically active iron chelator. Blood. (2009) 2:462–9. 10.1182/blood-2008-05-155952 18815282 PMC2615657

[49] Jiao Y Wilkinson J 4th Christine Pietsch E Buss JL Wang W Planalp R. Iron chelation in the biological activity of curcumin. Free Radic Biol Med. (2006) 7:1152–60. 10.1016/j.freeradbiomed.2005.11.003 16545682

[50] Yin H Zhou Y Wen C Zhou C Zhang W Hu X. Curcumin sensitizes glioblastoma to temozolomide by simultaneously generating ROS and disrupting AKT/mTOR signaling. Oncol Rep. (2014) 4:1610–6. 10.3892/or.2014.3342 25050915

[51] Chen TC Chuang JY Ko CY Kao TJ Yang PY Yu CH. AR ubiquitination induced by the curcumin analog suppresses growth of temozolomide-resistant glioblastoma through disrupting GPX4-Mediated redox homeostasis. Redox Biol. (2020) 30:101413. 10.1016/j.redox.2019.101413 31896509 PMC6940696

[52] Ferreira CA Ni D Rosenkrans ZT Cai W. Scavenging of reactive oxygen and nitrogen species with nanomaterials. Nano Res. (2018) 10:4955–84. 10.1007/s12274-018-2092-y 30450165 PMC6233906

[53] Su LJ Zhang JH Gomez H Murugan R Hong X Xu D. Reactive oxygen species-induced lipid peroxidation in apoptosis, autophagy, and ferroptosis. Oxid Med Cell Longev. (2019) 2019:5080843. 10.1155/2019/5080843 31737171 PMC6815535

[54] Sui X Zhang R Liu S Duan T Zhai L Zhang M. RSL3 drives ferroptosis through GPX4 inactivation and ROS production in colorectal cancer. Front Pharmacol. (2018) 9:1371. 10.3389/fphar.2018.01371 30524291 PMC6262051

[55] Wu W Wu Y Mayer K von Rosenstiel C Schecker J Baur S. Lipid peroxidation plays an important role in chemotherapeutic effects of temozolomide and the development of therapy resistance in human glioblastoma. Transl Oncol. (2020) 3:100748. 10.1016/j.tranon.2020.100748 32087559 PMC7033364

[56] Lo Dico A Salvatore D Martelli C Ronchi D Diceglie C Lucignani G. Intracellular redox-balance involvement in temozolomide resistance-related molecular mechanisms in glioblastoma. Cells. (2019) 8:1315. 10.3390/cells8111315 31653091 PMC6912456

[57] Zhu ZL Du SS Du YB Ren J Ying GG Yan Z. Glutathione reductase mediates drug resistance in glioblastoma cells by regulating redox homeostasis. J Neurochem. (2018) 1:93–104. 10.1111/jnc.14250 29105080

[58] Polewski MD Reveron-Thornton RF Cherryholmes GA Marinov GK Cassady K Aboody KS. Increased expression of system xc- in glioblastoma confers an altered metabolic state and temozolomide resistance. Mol Cancer Res. (2016) 12:1229–42. 10.1158/1541-7786.MCR-16-0028 27658422 PMC6237285

[59] Jiang L Kon N Li TY Wang SJ Su T Hibshoosh H. Ferroptosis as a p53-mediated activity during tumour suppression. Nature. (2015) 7545:57–62. 10.1038/nature14344 25799988 PMC4455927

[60] Wang H An P Xie E Wu Q Fang X Gao H. Characterization of ferroptosis in murine models of hemochromatosis. Hepatology. (2017) 2:449–65. 10.1002/hep.29117 28195347 PMC5573904

[61] Sehm T Rauh M Wiendieck K Buchfelder M Eyupoglu IY Savaskan NE. Temozolomide toxicity operates in a xCT/SLC7a11 dependent manner and is fostered by ferroptosis. Oncotarget. (2016) 46:74630–47. 10.18632/oncotarget.11858 27612422 PMC5342691

[62] Zhao HY Ji B Chen JG Huang QF Lu XG. Gpx 4 is involved in the proliferation, migration and apoptosis of glioma cells. Pathol Res Pract. (2017) 6:626–33. 10.1016/j.prp.2017.04.025 28552540

[63] Hangauer MJ Viswanathan VS Ryan MJ Bole D Eaton JK Matov A. Drug-tolerant persister cancer cells are vulnerable to GPX4 inhibition. Nature. (2017) 7679:247–50. 10.1038/nature24297 29088702 PMC5933935

[64] Fan Z Wirth AK Chen D Wruck CJ Rauh M Buchfelder M. Nrf2-Keap1 pathway promotes cell proliferation and diminishes ferroptosis. Oncogenesis. (2017) 8:e371. 10.1038/oncsis.2017.65 28805788 PMC5608917

[65] Yang WS Kim KJ Gaschler MM Patel M Shchepinov MS Stockwell BR. Peroxidation of polyunsaturated fatty acids by lipoxygenases drives ferroptosis. Proc Natl Acad Sci U S A. (2016) 34:E4966–4975. 10.1073/pnas.1603244113 27506793 PMC5003261

[66] Doll S Proneth B Tyurina YY Panzilius E Kobayashi S Ingold I. ACSL4 dictates ferroptosis sensitivity by shaping cellular lipid composition. Nat Chem Biol. (2017) 1:91–8. 10.1038/nchembio.2239 27842070 PMC5610546

[67] Yuan H Li X Zhang X Kang R Tang D. Identification of ACSL4 as a biomarker and contributor of ferroptosis. Biochem Biophys Res Commun. (2016) 3:1338–43. 10.1016/j.bbrc.2016.08.124 27565726

[68] Cheng J Fan YQ Liu BH Zhou H Wang JM Chen QX. ACSL4 suppresses glioma cells proliferation via activating ferroptosis. Oncol Rep. (2020) 1:147–58. 10.3892/or.2019.7419 31789401 PMC6912066

[69] Jung BJ Yoo HS Shin S Park YJ Jeon SM. Dysregulation of NRF2 in cancer: from molecular mechanisms to therapeutic opportunities. Biomol Ther (Seoul). (2018) 1:57–68. 10.4062/biomolther.2017.195 29212307 PMC5746038

[70] Taguchi K Yamamoto M. The KEAP1-NRF2 system in cancer. Front Oncol. (2017) 7:85. 10.3389/fonc.2017.00085 28523248 PMC5415577

[71] Xia M Yu H Gu S Xu Y Su J Li H. p62/SQSTM1 is involved in cisplatin resistance in human ovarian cancer cells via the Keap1-Nrf2-ARE system. Int J Oncol. (2014) 6:2341–8. 10.3892/ijo.2014.2669 25269472

[72] Zhu J Wang H Fan Y Lin Y Zhang L Ji X Targeting the NF-E2-related factor 2 pathway: a novel strategy for glioblastoma (review). Oncol Rep. (2014) 2:443–50. 10.3892/or.2014.3259 24926991

[73] Rocha CR Kajitani GS Quinet A Fortunato RS Menck CF. NRF2 and glutathione are key resistance mediators to temozolomide in glioma and melanoma cells. Oncotarget. (2016) 30:48081–92. 10.18632/oncotarget.10129 27344172 PMC5217002

[74] Shi L Li H Zhan Y. All-trans retinoic acid enhances temozolomide-induced autophagy in human glioma cells U251 via targeting Keap1/Nrf2/ARE signaling pathway. Oncol Lett. (2017) 3:2709–14. 10.3892/ol.2017.6482 28927033 PMC5588105

[75] Zhang L Wang H. FTY720 inhibits the Nrf2/ARE pathway in human glioblastoma cell lines and sensitizes glioblastoma cells to temozolomide. Pharmacol Rep. (2017) 6:1186–93. 10.1016/j.pharep.2017.07.003 29128799

[76] Kerins MJ Ooi A. The roles of NRF2 in modulating cellular iron homeostasis. Antioxid Redox Signal. (2018) 29:1756–73. 10.1089/ars.2017.7176 28793787 PMC6208163

[77] Sun X Ou Z Chen R Niu X Chen D Kang R. Activation of the p62-Keap1-NRF2 pathway protects against ferroptosis in hepatocellular carcinoma cells. Hepatology. (2016) 1:173–84. 10.1002/hep.28251 26403645 PMC4688087

[78] Roh JL Kim EH Jang H Shin D. Nrf2 inhibition reverses the resistance of cisplatin-resistant head and neck cancer cells to artesunate-induced ferroptosis. Redox Biol. (2017) 11:254–62. 10.1016/j.redox.2016.12.010 28012440 PMC5198738

[79] Ye P Mimura J Okada T Sato H Liu T Maruyama A Nrf2- and ATF4-dependent upregulation of xCT modulates the sensitivity of T24 bladder carcinoma cells to proteasome inhibition. Mol Cell Biol. (2014) 18:3421–34. 10.1128/MCB.00221-14 PMC4135628 25002527

[80] Chen D Rauh M Buchfelder M Eyupoglu IY Savaskan N. The oxido-metabolic driver ATF4 enhances temozolamide chemo-resistance in human gliomas. Oncotarget. (2017) 31:51164–76. 10.18632/oncotarget.17737 28881638 PMC5584239

[81] Chen Y Mi Y Zhang X Ma Q Song Y Zhang L. Dihydroartemisinin-induced unfolded protein response feedback attenuates ferroptosis via PERK/ATF4/HSPA5 pathway in glioma cells. J Exp Clin Cancer Res. (2019) 1:402. 10.1186/s13046-019-1413-7 31519193 PMC6743121

[82] Chen D Fan Z Rauh M Buchfelder M Eyupoglu IY Savaskan N. ATF4 promotes angiogenesis and neuronal cell death and confers ferroptosis in a xCT-dependent manner. Oncogene. (2017) 40:5593–608. 10.1038/onc.2017.146 28553953 PMC5633655

[83] Lee SW Kim HK Lee NH Yi HY Kim HS Hong SH. The synergistic effect of combination temozolomide and chloroquine treatment is dependent on autophagy formation and p53 status in glioma cells. Cancer Lett. (2015) 2:195–204. 10.1016/j.canlet.2015.02.012 25681668

[84] Shajani-Yi Z de Abreu FB Peterson JD Tsongalis GJ. Frequency of somatic TP53 mutations in combination with known pathogenic mutations in colon adenocarcinoma, non-small cell lung carcinoma, and gliomas as identified by next-generation sequencing. Neoplasia. (2018) 3:256–62. 10.1016/j.neo.2017.12.005 29454261 PMC5849803

[85] Blough MD Beauchamp DC Westgate MR Kelly JJ Cairncross JG. Effect of aberrant p53 function on temozolomide sensitivity of glioma cell lines and brain tumor initiating cells from glioblastoma. J Neuro Oncol. (2011) 1:1–7. 10.1007/s11060-010-0283-9 20593219

[86] Chiang MF Chou PY Wang WJ Sze CI Chang NS. Tumor suppressor WWOX and p53 alterations and drug resistance in glioblastomas. Front Oncol. (2013) 3:43. 10.3389/fonc.2013.00043 23459853 PMC3586680

[87] Wang X Chen JX Liu JP You C Liu YH Mao Q. Gain of function of mutant TP53 in glioblastoma: prognosis and response to temozolomide. Ann Surg Oncol. (2014) 4:1337–44. 10.1245/s10434-013-3380-0 24248532

[88] Tarangelo A Magtanong L Bieging-Rolett KT Li Y Ye J Attardi LD. p53 Suppresses metabolic stress-induced ferroptosis in cancer cells. Cell Rep. (2018) 3:569–75. 10.1016/j.celrep.2017.12.077 29346757 PMC5791910

[89] Zhou B Liu J Kang R Klionsky DJ Kroemer G Tang D Ferroptosis is a type of autophagy-dependent cell death. Semin Cancer Biol. (2019). 10.1016/j.semcancer.2019.03.002. [Epub ahead of print]. 30880243

[90] Hou W Xie Y Song X Sun X Lotze MT Zeh HJ. Autophagy promotes ferroptosis by degradation of ferritin. Autophagy. (2016) 8:1425–8. 10.1080/15548627.2016.1187366 27245739 PMC4968231

[91] Torii S Shintoku R Kubota C Yaegashi M Torii R Sasaki M. An essential role for functional lysosomes in ferroptosis of cancer cells. Biochem J. (2016) 6:769–77. 10.1042/BJ20150658 26759376

[92] Yang M Chen P Liu J Zhu S Kroemer G Klionsky DJ. Clockophagy is a novel selective autophagy process favoring ferroptosis. Sci Adv. (2019) 7:eaaw2238. 10.1126/sciadv.aaw2238 31355331 PMC6656546

[93] Liu J Kuang F Kroemer G Klionsky DJ Kang R Tang D. Autophagy-dependent ferroptosis: machinery and regulation. Cell Chem Biol. (2020) 27:420–35. 10.1016/j.chembiol.2020.02.005 32160513 PMC7166192

[94] Knizhnik AV Roos WP Nikolova T Quiros S Tomaszowski KH Christmann M. Survival and death strategies in glioma cells: autophagy, senescence and apoptosis triggered by a single type of temozolomide-induced DNA damage. PLoS One. (2013) 1:e55665. 10.1371/journal.pone.0055665 23383259 PMC3559438

[95] Buccarelli M Marconi M Pacioni S De Pascalis I D'Alessandris QG Martini M. Inhibition of autophagy increases susceptibility of glioblastoma stem cells to temozolomide by igniting ferroptosis. Cell Death Dis. (2018) 8:841. 10.1038/s41419-018-0864-7 30082680 PMC6079099

[96] Zheng DW Lei Q Zhu JY Fan JX Li CX Li C. Switching apoptosis to ferroptosis: metal-organic network for high-efficiency anticancer therapy. Nano Lett. (2017) 1:284–291. 10.1021/acs.nanolett.6b04060 28027643

[97] Lee YS Lee DH Choudry HA Bartlett DL Lee YJ. Ferroptosis-induced endoplasmic reticulum stress: cross-talk between ferroptosis and apoptosis. Mol Cancer Res. (2018) 7:1073–6. 10.1158/1541-7786.MCR-18-0055 29592897 PMC6030493

[98] Hong SH Lee DH Lee YS Jo MJ Jeong YA Kwon WT Molecular crosstalk between ferroptosis and apoptosis: emerging role of ER stress-induced p53-independent PUMA expression. Oncotarget. (2017) 70:115164–78. 10.18632/oncotarget.23046 PMC5777762 29383150

